# Involvement in decision-making processes and retention of health workers: findings from a cross-sectional study in the Rwandan Public District Hospitals

**DOI:** 10.11604/pamj.2019.34.129.16514

**Published:** 2019-11-05

**Authors:** Celestin Ndikumana, Ruth Tubey, Joshua Kwonyike

**Affiliations:** 1 College of Arts and Social Sciences, University of Rwanda, Kigali, Rwanda; 2 Institute of Development Studies, Moi University, Eldoret, Kenya; 3 School of Business and Economics, Moi University, Eldoret, Kenya

**Keywords:** Health workers, shared governance, merit pay process, systems for suggestions, intention to stay, district hospital, Rwanda

## Abstract

**Introduction:**

The contribution of the health workforce for better health care service provision is undoubtedly of great merit to any health system. However, the public district hospitals in Rwanda have been faced with the challenges of retaining the health personnel. This study looks into the management approach to address this challenge by investigating into the effect of employee involvement in the hospital decision-making processes on the retention of professional health workers.

**Methods:**

A cross-sectional design with quantitative approach was used. With a population of 469 health workers from 3 hospitals, a sample of 252 respondents was considered. Data collection was done by use of survey questionnaire. For data analysis, we used descriptive statistics to report perceived levels of involvement of health workers and intents to stay, and multiple logistic regression at 95% of confidence intervals to assess the effect of health workers? involvement in the hospital decision-making processes on the retention.

**Results:**

The findings revealed that health workers who perceived a high level of involvement in the hospital decision-making processes through the determination of teams for quality improvement in the health care service delivery were more likely to stay in the hospital (OR=100.111; P=0.001; CI=5.984-16.747) than those who perceived this function as low. It was also found that while an average level of involvement of health workers in the establishment of systems for suggestion in the hospital was associated with 6 odds of staying (OR=6.005; P=0.010; CI=1.529-23.571), health workers who perceived a high level of involvement were nearly 11 times more likely to stay (OR=10.952; P=0.001; CI=7.730-15.519) than their counterparts with low levels of perceptions.

**Conclusion:**

Although there are positive associations between involvement of health workers in the hospitals decision-making processes and the intentions to stay, the existing level of staff involvement may have a negative effect on retention capacity in the public district hospitals.

## Introduction

The outbreak of the 1994 genocide atrocities in Rwanda ravaged and almost destroyed the entire health system of the East African country [1]. In fact, while the disastrous events of the time left the country with almost no health infrastructure, the health workforce had dramatically reduced and the unavailability of health services in the entire country was noticed. During that time, for example, while there was only one doctor and one nurse serving the population of 16,001 and 1291 respectively as for 2012 [2], the midwifery career was still a bigger challenge as there was a count of 1 midwife for a population of 66,749 [3]. In the efforts to reinitiate health service delivery in the country, a new strategic policy to guide the reconstruction of the health system was issued in 1995 [4], which in the late years focused more on provision of adequate number of health workers [5-7]. This phase came with new strategies including training of health workers in order to address the shortage of the health workforce in the country [5], and the overall strategic orientation to improve the management of health institutions in general and the health personnel in particular [6]. The introduction of various strategies to address the issues of human resources for health in Rwanda, however, has not fully responded to the health capital challenges in the country. In fact, between 2010 and 2013 the rate of turnover was 11.3%, the figure which went up to 12.8% in 2014 [2]. The aim of this paper is to examine the human resource management approach to respond to the growing attrition of health workers in the country. The objectives of the study were, therefore, to determine the effect of exposure to shared governance in the health care institutions on retention of health workers, to examine the influence of staff involvement in determining teams for merit pay process in the hospital on the retention of health workers, to determine the effect of staff involvement in the determination of teams for quality improvement in health care service delivery on the retention of health workers, and to examine the influence of staff involvement in establishment of systems for suggestion in the hospital on retention of health workers.

As a general observation in the current situation of management of institutions, finding adequate employees is becoming a challenge [8]. As for that, it has been a priority to many organizations to turn their focus on retaining their workforce [9] by devising a number of strategies to make them remain in the work for a long period of time [10]. In the health care setting, a cosmopolitan belief is that retention of health workers is associated with better health outcomes [11], which calls for a number of strategies and policies to increase retention of health workers. In this view, involvement of health workers in health care institutions' decision-making processes has been considered as one of such strategies that respond to the issue of turnover of the health personnel [12]. In essence, employee involvement concerns the seeking of common interests for both employees and management, and in this perspective it is not only reflected in trade unions [13], but also it focuses on how employees participate in the institutional decision-making as a whole [14]. This results in the overall motivation of employees and reduces turnover. The concept of involvement in the health care setting is concerned with practices that lead to high levels of engagement of health workers through shared governance at different levels of the health care institution, health staff participation in nomination of teams for quality improvement in the health care service delivery, determination of merit pay processes in health institutions and health workers' consultation in the establishment of systems for suggestions in the health care institutions [15]. The role of employee involvement in decision-making on employee retention being extensively captured in the literature of human resource management [16-22], it emerges from different scholarly positions that the energy and commitment that employees bring and attach to the organization come from their different levels of involvement. As a result, the organization enjoys the reduced levels of turnover due to positive work environment resulting from involving health workers at different levels of decision-making processes. In the health care setting, involvement of the health personnel in decision-making becomes an opportunity for authority distribution and strengthening positive work environment in the health care institution as a long-term strategy, which operates at both individual and unit levels [23, 24]. As the core purpose of involving health workers in the decision-making processes concerns individual motivation which in turn leads to commitment towards the health care institution and health care service provision, it comes to the management to devise structural measures that lead to job satisfaction through the empowerment of health workers in order to boost their willing to remain in their work places [25].

In the study conducted by Hauck, Quinn and Fitzpatrick [12] and which sought to establish the associations between structural empowerment and turnover of nurses, the findings showed that the higher professional health workers are involved, the higher the feeling of job embeddedness and organizational commitment. The authors' conclusions are in agreement with the findings by Holton and O'Neill [26] whose study confirmed that health institutions with high involvement of health workers in the decision-making processes create positive work environment and subsequently record low rates intents to leave among different categories of the health personnel. Rondeau & Wagner [27] found in their study that involvement of nurses in decision-making processes is significantly associated with lower voluntary turnover. In the same way, health workers' involvement in decision-making processes as a way of fostering the improved work environment [28] results in satisfaction of the health personnel in general and through participative management practices. In addition, the decentralization of the decision-making in health care organizations leads to health workers' intent to stay [29, 30] and provides health institutions with high record of retention rates [31, 32].

## Methods

### Design and sampling

A cross-sectional design with quantitative approach was used. The study was conducted on the sample of 252 health workers selected from a population of 469 individuals including specialist and general doctors, nurses, midwives, pharmacists and dentists from three district hospitals. The category of health workers included in the study followed the International Standards Classification of Occupations [33]. In order to make sure the sample is inclusive of all categories of respondents, three major steps were followed. Firstly, based on their number and their expected their contribution to the study, all pharmacists and dentists were purposively included in the study. Secondly, by using a formula [34] the sample for the doctors, nurses and midwives was determined from the total number of these categories altogether. Thirdly, proportionate allocation was used to determine the number of participants in each hospital and in the categories of doctors, nurses and midwives. Simple random sampling was finally used to determine individual respondents among the sampled doctors, nurses and midwives in each hospital.

### Measurement, instrumentation and data collection

The variable of involvement in decision-making was measured through the four point likert-scale questionnaire with 12 items on health workers' degree of satisfaction with health workers' exposure to shared governance in the hospital, health workers' involvement in the creation of teams for quality improvement in the hospital, health workers' consultation in the determination of merit pay processes, and involvement of health workers in the establishment of systems for suggestion. The variable of retention was measured through health workers' intentions to stay in the health institution for the next three years. The four point likert scale was used to give respondents a room to provide definite choices to express their perceptions [35], and as for that the commonly used neutral response was removed from the traditional five-point likert scale as recommended by researchers on the human behavior [36].

### Data analysis

We used descriptive statistics to report demographic characteristics of respondents, the levels of perception on involvement of health workers in the hospital decision-making processes, and the current status of intentions to stay (or to leave) among health workers. In order to establish the effect of health workers' involvement in decision-making on retention, intention to stay was measured as a dichotomous outcome variable predicted by several independent variables. In this regard, multiple logistic regression was performed at 95% confidence intervals and 5% level of confidence. During the process of data analysis, variables of age, respondents' marital status, experience, education level and sex were controlled for by uploading them in the second model under the assumption that they have the potential to confound with the relationship between independent variables and the outcome variable. Results were reported using adjusted odds ratios in the adjusted multiple logistic regression model. All statistical analysis was performed using the STATA 13.1 software.

### Ethics

The ethical approval was provided by the University of Rwanda Institutional Review Board under the Ethical Clearance No 016RPGS017. The authorization to conduct the study in the 3 hospitals was also acquired from the Hospitals Research and Ethical Committees (1072/MSK/DH/2017; 247/HOP.KIBAG./2017; 483/KH/17). In addition, informed consent was sought from each individual respondent after detailed and clear explanation of the objectives and expected outcomes of the study.

## Results

### Characteristics of respondents

The social and demographic characteristics of respondents revealed that the age category of between 31 and 40 and that of between 41 and 50 make almost 75 percent of the total number of health workers with respective percentages of 37.04 percent and 37.86 percent. Married medical professionals who participated in the study accounted for 67.90 percent of the total number of respondents in this category. While most health professionals had a bachelor's degree as the highest level of education (56.38%), health workers with secondary education had the same count as those with PhD (1.23%). As far as the tenure is concerned, it was noticed that almost 60 percent of health workers who participated in the study had been in the health care service delivery profession for at least 6 years. While very few respondents had been health worker professionals for less than one year (1.47%), the range of 7 to 9 years of tenure counted for 14.40 percent of the total number of health workers. Concerning the time spent in the current health facility, all professional health workers had stayed for 9 years or less. It was also revealed that only 5.35 percent of respondents who participated in the study were foreign national health professionals (Table 1).

**Table 1:** Social and demographic characteristics of respondents

Gender	Count and percentage
Male	129 (53.09)
Female	114 (46.91)
Age of respondents	
30 years and below	43 (17.70)
Between 31-40 years	90 (37.04)
Between 41-50 years	92 (37.86)
51 years +	18 (7.41)
Marital status	
Single	65 (26.76)
Married	165 (67.90)
Divorced	8 (3.29)
Separated	3 (1.23)
Widowed	2 (0.82)
Level of education	
Secondary education (A2)	3 (1.234)
University Diploma (A1)	83 (34.16)
Bachelor’s Degree (A0)	137 (56.38)
Masters’ Degree	17 (7.00)
PhD	3 (1.23)
Experience in the health care service delivery	
Below 1 year	6 (2.47)
Between 1 and 3 years	67 (27.57)
Between 4 and 6 years	74 (30.45)
Between 7 and 9 years	53 (21.81)
Between 7 and 9 years	8 (3.29)
10 years and more	35 (14.40)
Time spent in the current hospital	
Below 1 year	29 (11.93)
Between 1 and 3 years	82 (33.74)
Between 4 and 6 years	106 (43.62)
Between 7 and 9 years	26 (10.70)
Between 7 and 9 years	0 (0.00)
10 years and more	0 (0.00)
Nationality	
Rwandan	230 (94.65)
Non-Rwandan	13 (5.35)
Total (n) 243 (100.00)	

### Professional health workers' intentions to stay

The study findings show that while 51.44 percent of health workers had intentions to stay, their counterparts with intentions to leave counted for 49.56 percent. Within categories of respondents, a big count of health workers who were willing to stay was found among doctors (67.35%). In the same viewpoint, slightly more than half of nurses and midwives had no intentions to stay, the rate which respectively increased up to 68 percent and 68.75 percent among pharmacists and dentists (Table 2).

**Table 2:** Intentions to stay among professional health workers

Intentions to stay	Doctors	Nurses and midwives	Pharmacists	Dentists	Total
With no intentions to stay	32.65	48.37	68.00	68.75	48.56
With intentions to stay	67.35	51.63	32.00	31.25	51.44

### Perceived level of health workers' involvement in the hospitals decision-making processes

The study investigated health workers' perceived levels of involvement in the health care institutions decision-making processes. While majority of dentists, pharmacists and nurses (and midwives) perceived a low level of health workers' exposure to shared governance (68.765%, 60% and 55.56% respectively) in the hospitals, most doctors perceived that there was an average level involvement of health workers in the hospital decision-making processes through exposure to shared governance (51.02%). The highest perceived level of health workers' exposure to shared governance in their respective work places was recorded among less than 20 percent across all categories of respondents. An average perceived level of health workers' involvement in determining teams for quality improvement in the hospitals outweighed among doctors (51.02%), nurses and midwives (44.44%), and dentists (48.00%). While involvement of health workers in determining the teams for quality improvement was perceived as low and average at the same rate among pharmacists (37.50%), 26.45 percent of doctors were of the perception involvement of health workers in the determination of teams for quality improvement in the health care service delivery was high. Nurses (and midwives) recorded the biggest number of participants who perceived a low level of involvement of health workers in the determination of teams for quality improvement in the hospital (30.07%). Majority of respondents perceived a very low level of consultation of health workers for merit pay processes in health care institutions: 69.39 percent of doctors, 60.13 percent of nurses and midwives, and 60 percent of dentists and 75 percent of pharmacists. As for the involvement of health workers in the establishment of systems for suggestions in the health care institutions, majority of the dentists perceived this function to be at an average level (48.00%). The perceived low level of involvement of health workers in such systems was predominantly recorded among doctors (48.98%), nurses and midwives (41.83%) and pharmacists (68.75%) (Table 3) Annex 1.

**Table 3:** Perceived level of involvement and participation in decision-making

	Area of work				
	Doctors (N=49)	Nurses/Midwives (N=153)	Dentists (N=25)	Pharmacists (N=16)	Total (N=243)
Exposure to shared governance					
Low	26.53	55.56	60.00	68.75	51.03
Average	51.02	33.33	28.00	12.50	34.98
High	22.45	11.11	12.00	18.75	13.99
Teams for quality improvement					
Low	22.45	30.07	24.00	37.50	28.40
Average	51.02	44.44	48.00	37.50	45.68
High	26.45	28.00	28.00	25.00	25.93
Consultation for merit pay process					
Low	69.39	60.13	60.00	75.00	62.96
Average	8.16	28.10	28.00	18.75	23.46
High	22.45	11.76	12.00	6.25	13.58
Establishment of systems for suggestions					
Low	48.98	41.83	36.00	68.75	44.44
Average	28.57	39.22	48.00	6.25	35.80
High	22.45	18.95	16.00	25.00	19.75
Overall involvement in decision-making					
Low	30.61	39.22	24.00	68.75	37.86
Average	46.94	35.95	60.00	12.50	39.09
High	22.45	24.84	16.00	18.75	23.05

**Annex 1:** Questionnaire to assess the perceived level of involvement of health workers in the hospital decision-making processes. Strongly agree=4; agree=3; disagree=2; strongly disagree=1

S/N	Statement	4	3	2	1
	Exposure to shared governance				
1	Professional health workers are consulted for the views aiming at solving problems which are in the hospital				
2	Employees are consulted for views on decisions regarding their work lives				
	Teams for quality improvement				
3	Professional health workers are given opportunities to meet in groups in order to identify, analyze and solve work-related problems pertaining their job				
4	Professional health workers from different departments are facilitated to meet (under supervision of management) in order to improve on the common goals (like provision of quality of health care)				
5	Employee are grouped in self-directed teams (without supervision of management) for improvement on common goal (like provision of quality of health care)				
6	Decision from these meetings are considered by management and administration of the hospital				
	Consultation for merit pay processes				
7	Hospital management and administration provides necessary support for self-directed teams (internal rules and regulations, mission and vision of the hospital, policies and procedures) for their improvement				
8	Professional health workers in this hospital take part in determination of their colleagues who should receive merit pay				
9	Professional health workers take part in deciding the amount of merit pay to be given to a colleague in the case such a practice is to happen at the hospital				
	Establishment of systems for suggestion				
10	Work councils for professional health workers have effective channels to address professional health workers’ challenges				
11	Professional health workers’ council suggestions are understood and considered by hospital management				
12	Work councils are considered as good organs for protection of professional health workers’ rights				

### Involvement in the hospitals decision-making processes and intentions to stay

In essence, the aim of the study was to establish the effect of involvement of the health workers in the hospital decision-making processes on the retention. The data on health workers' exposure to shared governance, health workers’ involvement in the determination of teams for quality improvement, health workers' consultation for merit pay processes, their involvement in the establishment of systems for suggestion and intentions to stay were subjected to a multiple logistic regression model in order to observe the associations between predictors and the outcome variable (Table 4). While all predictors were associated with health workers intentions to stay in the unadjusted model, accounting for potential confounding factors showed that involvement of health workers in the determination of teams for quality improvement and establishment of systems for suggestions in the hospitals was associated with intentions to stay. In fact, health workers who perceived a high level of involvement in the hospital decision-making processes through the determination of teams tor quality improvement in the health care service delivery were more (OR=100.111; P=0.001; CI=5.984-16.747) than those who perceived this function as low. It was also found that health workers who perceived that involvement in terms of establishment of systems for suggestion was at average level in the hospitals were 6 times more likely to stay (OR=6.005; P=0.010; CI=1.529-23.571) than those who perceived this practice as low. Similarly, nearly 11 odds of staying were recorded among health professionals whose perceptions on the level of involvement of health workers in the hospital decision-making rated the establishment of systems for suggestion as high (OR=10.952; P=0.001; CI=7.730-15.519). Other factors that were found to be associated with intentions to stay included having a masters and a PhD (OR=11.027; P=0.015; CI=1.584-76.726), being a nurse or a midwife (OR=0.151; P=001; CI=0.047-0.484), being a pharmacist or a dentist (OR=0.026; P<0.001; CI=0.004-0.161), having stayed in the health care service for 8 years and more (P=14.757; P0.033; CI=1.239-17.571), having stayed in the current hospital for 3 years and more (OR=0112; P=0.004; CI=0.025-0.497), and being a foreign national (OR=0.69; P=0.023; CI=0.006-0.694).

**Table 4:** Involvement in decision-making and intentions to stay

	Model 1				Model 2			
Intentions to stay	OR	95 % CI		P>[z]	OR	95 % CI		P>[z]
Shared governance								
Low	Ref.				Ref.			
Average	2.709	1.191	6.163	0.017	1.686	0.536	5.304	0.371
High	0.658	0.088	4.919	0.684	0.258	0.015	4.324	0.347
Quality improvement								
Low	Ref.							
Average	0.227	0.100	0.5130	0.000	1.660	0.396	6.953	0.488
High	3.942	0.567	27.393	0.165	100.111	5.984	16.747	0.001
Merit pay process								
Low	Ref.				Ref.			
Average	0.227	0.100	0.5130	0.000	3.529	0.929	13.404	0.064
High	3.942	0.567	27.393	0.165	0.217	0.016	2.929	0.250
Systems for suggestion								
Low	Ref.				Ref.			
Average	3.665	1.385	9.692	0.009	6.005	1.529	23.571	0.010
High	24.914	3.378	183.709	0.002	10.952	7.730	15.519	0.001
Gender								
Male					Ref.			
Female					0.985	0.361	2.684	0.977
Age group								
30 and below					Ref.			
31-45					1.371	0.293	6.408	0.688
46+					0.256	0.041	1.585	0.143
Marital status								
Single					Ref.			
Married					0.332	0.085	1.291	0.112
Ever married					1.149	0.088	14.864	0.915
Level of education								
Diploma or less					Ref.			
Bachelor’s Degree					1.114	0.439	2.825	0.819
Others					11.027	1.584	76.726	0.015
Area of work								
Doctors					Ref.			
Nurses and midwives					0.151	0.047	0.484	0.001
Others					0.026	0.004	0.161	0.000
Experience in service								
Less than 3 years					Ref.			
3-7 years					4.093	0.600	27.910	0.150
8 years +					14.757	1.239	17.571	0.033
Experience in facility								
Less than 3 years					Ref.			
3 years and more					0.112	0.025	0.497	0.004
Nationality								
Rwandan					Ref.			
Non-Rwandan					0.069	0.006	0.694	0.023

## Discussion

The study findings showed that in general intentions to stay are of unsatisfying level among professional health workers in the study setting. Intentions to stay being a major predictor of retention of health workers, the study findings constitute a basis to confirm the existing pattern of movement of health workers in the sub-Saharan African countries as it was found out in studies conducted inGhana [37], Malawi [38] and Kenya [39]. Needless to say, the existing belief is that the category of health workers that includes doctors would be with high intentions to leave compared with other categories of the health personnel. However, the study findings show that a high rate of intentions to stay was recorded among medical doctors. Such a pattern was similarly found in the study conducted in Malawi [40] and it was justified by the fact that health workers with low qualifications were attracted by salaries in the non-governmental organizations and subsequently had high levels of intentions to leave. For this study, the fact that health workers in the category of medical doctors perceived a high level of health workers' involvement and participation in the hospital decision-making processes through their exposure to shared governance may explain their recorded high rates of intents to stay in comparison with other categories of health workers. This may be linked to the fact that medical doctors occupy high positions and are part of the decision-making bodies in the hospitals. Although involvement of health workers in the hospital decision-making processes is an important practice in the governance of health care institutions [41] and a motivating factor contributing to retention of the health personnel [15], the study showed that by considering all categories of health workers who participated in the study, the level of involvement was not satisfying in general. The study findings show that intentions to stay among professional health workers are affected by a number of variables of involvement in decision-making as a retention strategy, as it was demonstrated by other studies examining predictors of retention of health workers [41, 42]. The findings are also in conformity with results of the study conducted by Rondeau [15] which came up with conclusions that high involvement of health workers is associated with lower turnover among nurses. Similarly, the findings from a study which investigated the role of governance through a review of case studies in low and middle income countries [43] showed that health workers' involvement at different levels of decision-making at both individually level and through the unions should be of considerable focus in the management of health care institutions. Such a practice is necessary in order to avoid non-participative policy formulation which may meet resistance and consequently affect retention of health workers in the future. In addition, the multifactorial nature of determinants of retention is validly extended to the values of empowerment and trust in the hospitals as the major health care institutions. Such values were explored in the study which used a systematic review method to investigate the factors that affect retention of nurses [44] and found that such practices increase motivation of health workers and lead to the overall reduced turnover among these categories of the health personnel. This study having considered three district hospitals from the capital city of the country is limited by the generalization of the findings to other health care institutions, although the sample was highly representative of the population where the study was conducted. For this reason, it is understood a survey with a larger sample of respondents and from other areas could lead to a further and deep analysis to understand the phenomenon of retention of health workers. In addition to this, the study having investigated the involvement of health workers in the hospitals decision-making processes and its influence on their retention, it focused on the employees who were employed in the health care institutions at the time when the survey was conducted. Hence the variable of retention was measured by health workers' intentions to stay as a major predictor of retention. It is therefore understood that the study was limited by the fact that it did not include health workers who had left the institutions in order to understand their views on major causes of turnover and turnover intentions among professional health workers.

## Conclusion

Based on the findings of the study, it was concluded that involvement of health workers in the health care institution decision-making process is a key retention the health personnel. It could be revealed, however, that the current level of involvement of professional health workers in the hospitals' decision-making processes may negatively affect the health professionals' intentions to stay and as for that adversely have an effect on retention of health workers in the public district hospitals.

### What is known about this topic

- Health workers are part of health system and they are foundation of better health care service delivery;
- There is a challenge of retaining the health workforce in developing countries, including Rwanda;
- Involvement of health workers in health care institutions' decision-making processes is a determining factor for the retention of the health personnel.

### What this study adds

- The status of intentions to stay as a major predictor of retention of health workers in the public hospitals in Rwanda; the rate of intentions to stay is considerably low;
- The level of involvement of health workers in health care institutions' decision-making processes in the Rwandan public district hospitals is not at satisfying;
- The study provides the insights on how a human resource management approach can benefit healthcare institutions by boosting retention through involvement of health workers at different levels of the decision-making.

## Competing interests

The authors declare no competing interests.
